# Cross-Linked Starch as Media for Crystal Violet Elimination from Water: Modeling Batch Adsorption with Fuzzy Regression

**DOI:** 10.3390/molecules29163894

**Published:** 2024-08-17

**Authors:** Mehdi Bahrami, Mohammad Javad Amiri, Rosa Busquets, Mohammad Javad Nematollahi

**Affiliations:** 1 Department of Water Science and Engineering, Faculty of Agriculture, Fasa University, Fasa 74616-86131, Iran; 2 Research Institute of Water Resources Management in Arid Region, Fasa University, Fasa 74616-86131, Iran; 3 Department of Civil, Environmental and Geomatic Engineering, University College London, Gower St., Bloomsbury, London WC1E 6BT, UK; r.busquets@kingston.ac.uk; 4 Faculty of Health, Science, Social Care and Education, School of Pharmacy and Chemistry, Kingston University, Penrhyn Road, Kingston Upon Thames KT1 2EE, UK; 5 Department of Geology, Faculty of Sciences, Urmia University, Urmia 57561-51818, Iran; mj.nematollahi@urmia.ac.ir

**Keywords:** dye, sorbent, starch, fuzzy regression, wastewater treatment, 6th SDG

## Abstract

A scalable and cost-effective solution for removing pollutants from water is to use biodegradable and eco-friendly sorbents that are readily available such as starch. The current research explored the removal of crystal violet (CV) dye from water using chemically modified potato starch. The adsorbent was prepared by cross-linking potato starch with sodium trimetaphosphate (STMP). The impact of various operating factors including pH, temperature, contact time, initial CV concentration, and adsorbent dosage on the removal of CV were investigated using batch experiments. The adsorption data were analyzed using a fuzzy regression approach, which provided a range-based representation of the model’s output. The cross-linked starch adsorbent was mesoporous, with a mean pore diameter of 9.8 nm and a specific surface area of 2.7 m^2^/g. The adsorption of CV by the STMP cross-linked potato starch was primarily influenced by the adsorbent dosage, followed by the solution pH, temperature, initial CV concentration, and contact time. The fuzzy regression model accurately predicted the independent experimental data of CV removal with an R^2^ of 0.985, demonstrating its value as a tool for the continuous monitoring of CV removal as well as optimizing water treatment conditions.

## 1. Introduction

Synthetic dyes have become increasingly popular alternatives to natural dyes due to their lower cost, improved colorfastness, and ease of application [1]. Some commonly used synthetic dyes are non-biodegradable and have carcinogenic properties, making them hazardous to the ecosystem. Consequently, their presence in water bodies can lead to detrimental effects on vegetation, human health, and wildlife [1,2]. Growing environmental and public health concerns have created an urgency to develop effective treatment methods to mitigate the adverse impacts of synthetic dye-contaminated effluents on the environment and public well-being [2,3,4,5,6]. Synthetic dyes are classified as cationic, anionic, and non-ionic dyes, with cationic dyes presenting a higher risk compared to the other types [7].

Among the cationic dyes, crystal violet (CV), belonging to the triphenylmethane structural class [8], is a prominent pollutant found in industrial wastewater discharges from various sectors including color paints, pharmaceuticals, leather, detergents, fertilizers, varnishes, and waxes [9]. Exposure to CV can lead to severe health issues in humans including skin and eye irritation, nausea, weight loss, kidney diseases, gastrointestinal and respiratory tract irritation, adverse reproductive effects, and an increased risk of cancer [10,11]. Various approaches have been explored for the removal of CV from wastewater including photocatalytic degradation [12], chemical oxidation [13], electrochemical treatment [14], ozonation [15], membrane filtration [16] as well as adsorption processes [17,18,19,20,21]. Of these approaches, adsorption has emerged as a highly promising technique for removing CV from water bodies due to its simplicity, ease of operation, cost-effectiveness, efficiency, and eco-friendly nature [17,18,19,20,21]. Mecheri et al. [17] synthesized a kaolinite–cellulose composite for the removal of CV and reported a maximum adsorption capacity of 294.12 mg/g. A study by Thamer et al. [18] focused on the development of a biocomposite hydrogel for the removal of CV from water. The researchers incorporated activated carbon derived from pomegranate peels into tragacanth gum to create the biocomposite material. Using the Sips adsorption model, the researchers determined that the maximum adsorption capacity of the PPAC/TG biocomposite was 455.61 mg/g at a temperature of 25 °C. In a study by Mosoarca et al. [19], the researchers utilized motherwort (*Leonurus cardiaca* L.) biomass as an adsorbent for the removal of crystal violet dye from aqueous solutions. The kinetic data analysis revealed that the adsorption process reached equilibrium within 50 min. Furthermore, the researchers employed the Taguchi method and ANOVA analysis to determine the most influential parameter in the adsorption process, which was found to be the solution pH.

Starch can be used to make bio-based adsorbents. Starch is the second most abundant renewable and biodegradable resource after cellulose [22]. Native starch granules have semi-crystalline structures, composed of two fundamental glucosidic macromolecules: amylose and amylopectin. Amylose consists of linear molecules of glucose units linked primarily by 99% (1–4) α-D-glycosidic bonds, with only a small fraction of 1% (1–6) α-linkages [23]. In contrast, amylopectin is a highly branched polymer, containing approximately 95% α (1–4) linkages and 5% α (1–6) linkages [24]. This complex and well-defined structure of starch is a key factor that enables its versatile applications, particularly in the context of pollutant removal from wastewater. However, the poor characteristics of native starch such as its tendency to retrograde, hydrophobic nature, poor pollutant uptake capacity, and low tensile strength limit its direct use in practical applications [25]. To overcome the limitations of native starch and fully utilize its potential as a versatile bio-based adsorbent, various chemical modifications such as esterification, cross-linking, oxidation, and acid hydrolysis have been performed to incorporate active functional groups [2,25,26].

For instance, Amiri et al. [2] synthesized starch nanocrystals via acid hydrolysis to eliminate the amorphous regions of native corn starch and evaluated their adsorption capacity for methylene blue removal. The results indicated that the maximum adsorption capacity was increased from 63 mg/g for native corn starch to 79.95 mg/g for the starch nanocrystals. Bahrami et al. [27] synthesized potato phosphate starch via phosphorylation. The results indicated that the maximum lead removal of 99.9% was achieved at an initial lead concentration of 63.57 mg/L, a pH of 5, and a reaction time of 10 min. Tan et al. [4] synthesized a magnetic starch-based adsorbent for the removal of CV, which allowed for easy separation and recycling. The maximum adsorption capacity of the adsorbent was achieved at 49 mg/g. Yeamin et al. [28] developed a polymeric adsorbent by integrating polyaniline with starch. The findings revealed that the resulting polyaniline/starch adsorbent exhibited a reduced particle size and superior adsorption efficiency for methylene blue and orange green dyes in comparison to other starch-based adsorbents.

Cross-linking starch chemically has emerged as a highly effective method that enhances its physicochemical properties, outperforming alternative approaches. Crosslinking involves the formation of intra- and inter-molecular bonds within the starch molecule, which improves its properties [26,29]. The effectiveness of starch cross-linking is contingent upon several key variables including the origin of the starch, the concentration of the cross-linking agent employed, the extent of substitution, and the specific parameters of the reaction process such as pH, the duration of the reaction, and the temperature of the process. Among the cross-linking reagents, sodium trimetaphosphate (STMP) stands out due to its notable advantages including non-toxicity, ease of application, relatively low cost, slower penetration rate, and the ability to facilitate crosslinking reactions without the need for additional catalysts [29,30].

While starch-based adsorbents possess many desirable properties for water treatment, the adsorption process itself presents inherent challenges that make it difficult to develop accurate estimation models suitable for all environments. The adsorption process is often characterized as incidental, nonlinear, and complex, with a high degree of uncertainty [31,32,33]. These inherent complexities of the adsorption process make it challenging to derive a single, universal formula that can accurately estimate the removal of target pollutants across diverse environmental conditions. Traditional modeling techniques may struggle to capture the nuances and uncertainties associated with adsorption processes, leading to inaccuracies when applied in different settings. To address the limitations of traditional modeling techniques, researchers are exploring the use of intelligent soft computing methods such as fuzzy regression as a new avenue for developing more robust and adaptable models for adsorption processes [34]. Fuzzy regression techniques can handle the nonlinearity, complexity, and uncertainty inherent in adsorption systems. By incorporating fuzzy logic and regression analysis, these methods can better account for the ambiguity and imprecision in the relationships between adsorbent properties, environmental factors, and pollutant removal efficiency. The primary goal of this research was to examine cross-linked starch derived from potato starch using STMP and evaluate its potential to remove CV from aqueous solutions. The study specifically aimed to use fuzzy regression modeling to identify the key parameters influencing the adsorption capacity of cross-linked starch for removing CV. These parameters include the initial dye concentration, adsorbent dose, pH, contact time, and temperature. By employing fuzzy regression analysis, this study aimed to address the existing research gap and supply practical prospects for the potential of this soft computing technique for modeling the complex adsorption process.

## 2. Results and Discussion

### 2.1. Sorbent Characteristics

**Figure 1:**
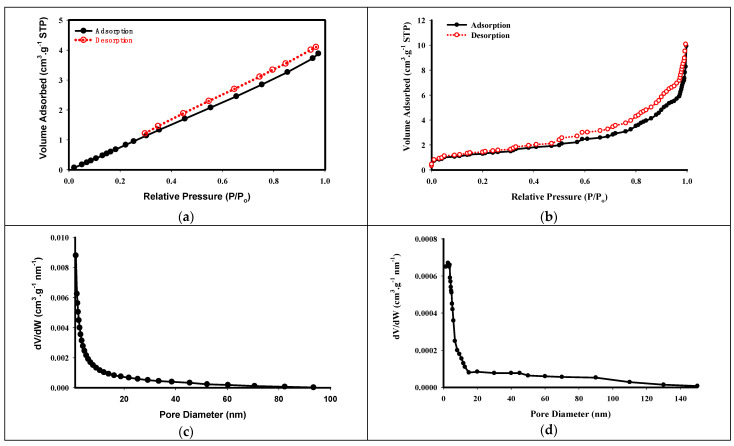
Nitrogen adsorption–desorption isotherms of (**a**) native potato starch and (**b**) STMP cross-linked starch as well as the pore size distribution of (**c**) native potato starch and (**d**) STMP cross-linked starch.

Figure 1 illustrates the nitrogen (N_2_) adsorption–desorption isotherms of the native potato starch as well as the STMP cross-linked starch, along with their corresponding pore size distribution. The adsorption isotherm patterns exhibited by the native potato starch and the STMP cross-linked starch differed significantly, reflecting the impact of the modification process on the adsorption behavior. For the native potato starch, the adsorption isotherm pattern could be categorized as linear, indicating a relatively constant adsorption capacity across the range of studied concentrations. This linear isotherm behavior is often associated with adsorbents possessing a homogeneous surface and a limited number of adsorption sites (Figure 1a). In contrast, the adsorption isotherm pattern of the STMP cross-linked starch obeyed an IV-type isotherm, characterized by an initial horizontal plateau followed by a steep uptake in adsorption capacity at higher relative pressures (Figure 1b). This type of isotherm is typically observed in mesoporous adsorbents with pore diameters ranging from 2 to 50 nanometers. Furthermore, the STMP cross-linked starch exhibited an H3-type hysteretic loop in its adsorption–desorption isotherm. This hysteresis loop is associated with adsorbents containing slit-shaped pores or exhibiting non-rigid behavior such as swelling or contraction during adsorption and desorption processes.

The analysis of the experimental data provides further insights into the impact of the STMP cross-linking modification on the porous structure of the starch adsorbent. The findings revealed that the average pore diameter of the native potato starch was estimated to be 7.2 nm (Figure 1c), which was lower than the average pore diameter of 9.8 nm obtained for the STMP cross-linked starch (Figure 1d). This increase in the average pore diameter of the cross-linked starch can be attributed to the structural changes induced by the cross-linking process. The introduction of cross-links within the starch matrix can lead to the formation of a more open and porous structure, resulting in an increased average pore size. The Brunauer–Emmett–Teller (BET) analysis indicated a specific surface of 2.7 m^2^/g for the STMP cross-linked starch, which was higher than the value of 2.2 m^2^/g obtained for the native potato starch. Moreover, the pore volume of STMP cross-linked starch was measured to be 0.009 cm^3^/g.

**Figure 2:**
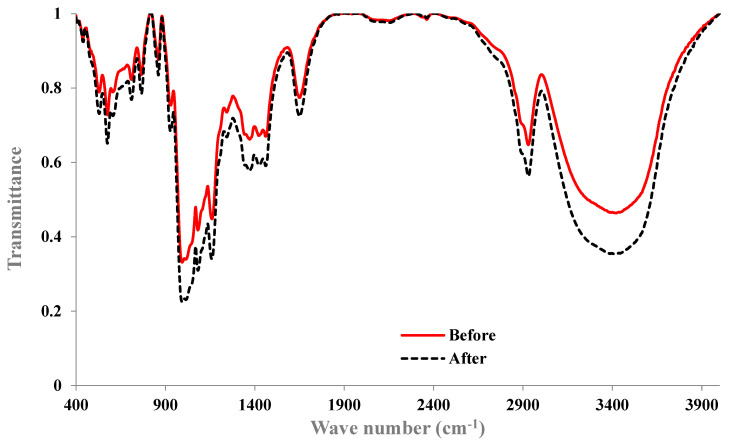
FTIR spectra of STMP cross-linked starch before and after the adsorption of crystal violet.

The FTIR spectra of the cross-linked potato starch before and after CV adsorption are shown in Figure 2. An increase in peak intensity after adsorption suggests that the functional group is involved in binding with the dye. In the spectra, wave numbers between 3200–3600 cm^−1^ were attributed to the existence of O–H bonds. The bands marked in the 2800–3000 cm^−1^ region were associated with C–H bending vibrations, and the peaks at 2930 and 3000 cm^−1^ resulted from C–H stretching. Intramolecular hydrogen bonds resulted in prominent peaks at 1605, 1651.7, and 1844.6 cm^−1^, indicating C=O stretching. Additionally, peaks at 1140.7, 1152.3, 1237.1, and 1252.5 cm^−1^ were related to C–O. The absorption peaks characteristic of P–O–C (96.4, 1071.3, and 1088.6 cm^−1^) appeared in the spectra of the cross-linked starch samples because of the presence of cross-linking agents. The wave numbers 884, 861, and 824.4 cm^−1^ are typically associated with C–O–C stretching, C–H bending, and glycosidic linkage vibrations, respectively [30,35]. This analysis proves the existence of functional groups contributing to adsorption like O–H, C–H, C=O, and P–O–C.

**Figure 3:**
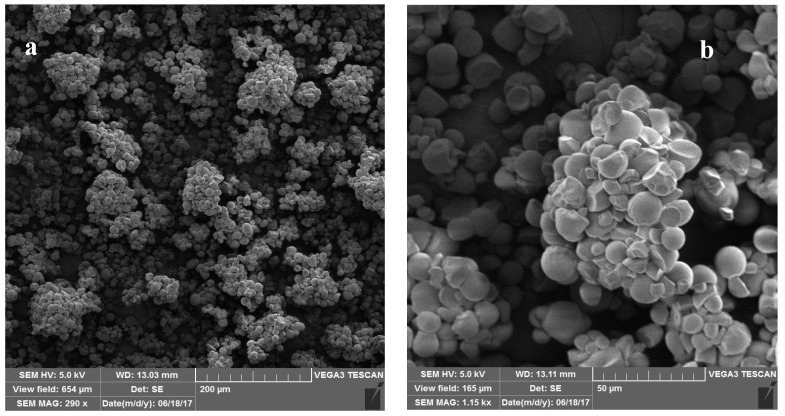

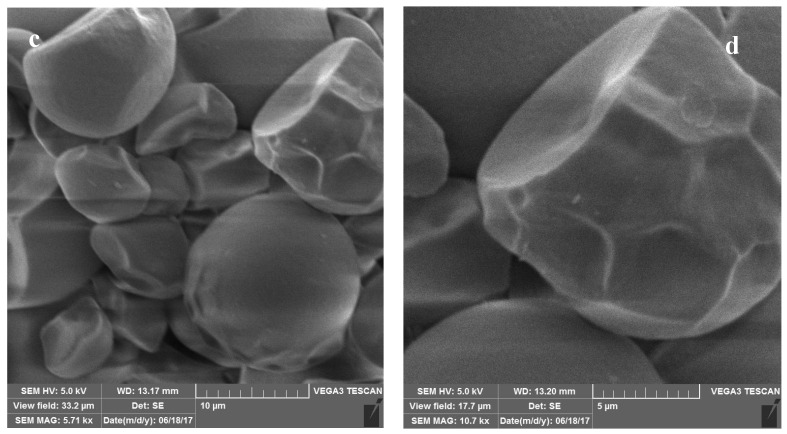
Scanning electron micrographs of STMP cross-linked starch in (**a**) 200 μm, (**b**) 50 μm, (**c**) 10 μm, and (**d**) 5 μm magnifications.

Figure 3 displays the scanning electron micrographs of cross-linked potato starch at magnifications of 5, 10, 50, and 200 μm. The results showed that this material has a dispersed texture with spherical and polyhedral shapes. Through the SEM analysis, it was determined that the average pore diameter of the synthesized cross-linked starch was approximately 9.8 nm.

**Figure 4:**
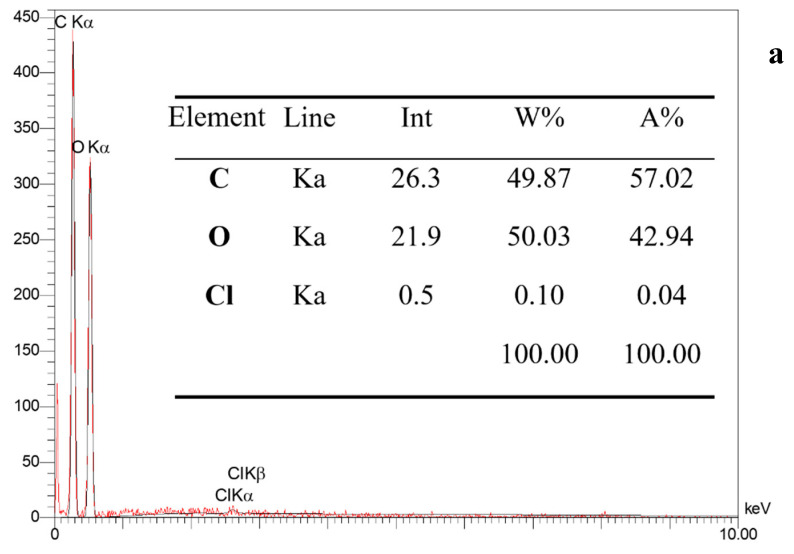

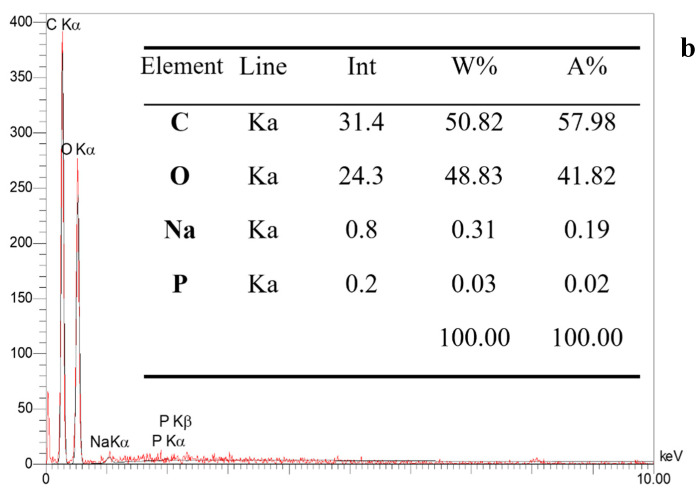
Energy-dispersive X-ray spectroscopy of potato starch (**a**) and STMP cross-linked potato starch (**b**).

The EDX patterns of the potato starch powder and synthesized cross-linked starch (Figure 4) revealed that the main elements present in native starch were carbon and oxygen. Additionally, the presence of Na and P signals on the modified starch surface was attributed to the STMP agent. These EDX patterns offer valuable insights into the elemental composition and verify the successful modification of the starch using STMP.

**Figure 5:**
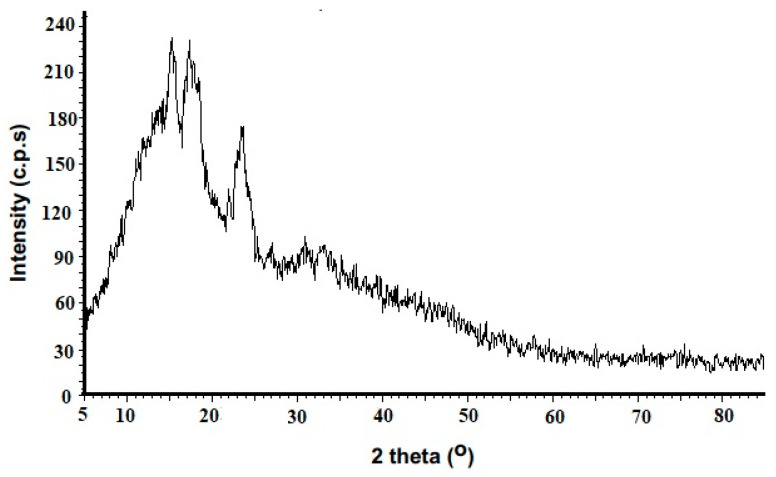
X-ray diffraction pattern of STMP cross-linked potato starch.

The X-ray diffraction pattern of cross-linked potato starch is shown in Figure 5. The XRD pattern of modified starch indicates that it had a less crystalline structure. The results revealed the presence of three characteristic peaks for cross-linked potato starch at 2θ values of 15.181°, 17.05°, and 23.07°. Additionally, Figure 5 demonstrates that the modified starch showed a typical A-type XRD pattern. Similar findings have also been documented by reference [27].

### 2.2. Evaluation of Effective Adsorption Parameters

#### 2.2.1. Impact of pH

**Figure 6:**
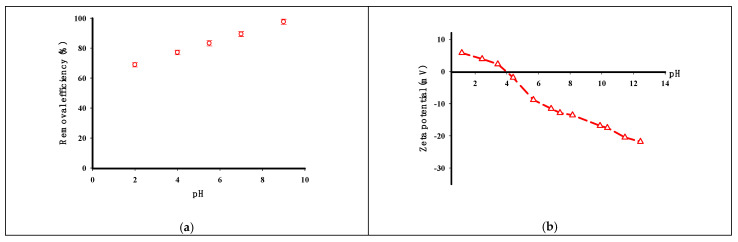
The impact of pH on the elimination efficiency of CV by the STMP cross-linked potato starch (initial concentration 100 mg/L, adsorbent dose 0.2 g, time 90 min, temperature 30 °C, volume of solution 200 mL) (**a**) and zeta potential of the STMP cross-linked potato starch at diverse pH values (**b**).

According to Figure 6a, there was an increase in the solution’s pH, resulting in a higher percentage of CV dye removal. The maximum elimination of 97.55%, was achieved at a pH of 9. According to [36,37], the estimated pK_a_ values of crystal violet is 5.31 and 8.64. At acidic pH, the dominant species is the protonated form (CV^+^); at neutral pH, both CV^0^ and CV^+^ exist; and at alkaline (basic) pH, CV^+^ accepts a hydroxyl to form the neutral CV molecule. The analysis of the point of zero charge (pH_PZC_) for the STMP cross-linked potato starch, as depicted in Figure 6b, revealed a crucial parameter influencing the adsorption behavior of the cationic dye crystal violet (CV). The pH_PZC_ value was determined to be 3.96, indicating that below this pH, the STMP cross-linked potato starch surface carried a net positive charge, while above a pH of 3.96, the surface acquired a net negative charge. At solution pH values below the pH_PZC_ of 3.96, both the STMP cross-linked potato starch and the CV dye molecules had positive charges. Consequently, electrostatic repulsion forces arose between the positively charged adsorbent surface and the positively charged CV dye molecules, hindering the adsorption process and potentially leading to a reduced adsorption capacity. Conversely, when the solution pH was raised above the pH_PZC_ value of 3.96, the STMP cross-linked potato starch surface became negatively charged. This negative surface charge facilitated favorable electrostatic interactions with the positively charged CV dye molecules, thereby enhancing the adsorption tendency and potentially increasing the overall adsorption capacity of the adsorbent. 

#### 2.2.2. Impact of Contact Time

**Figure 7:**
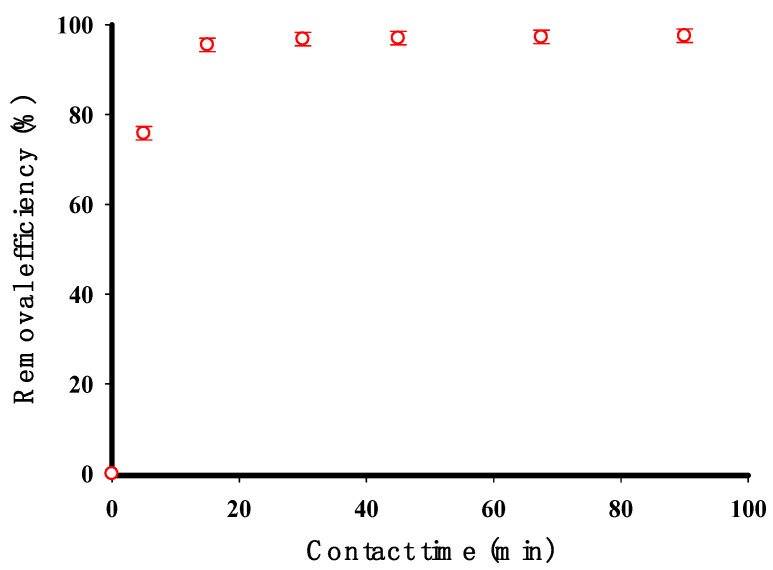
The contact time impact on the removal efficiency of CV by the cross-linked potato starch (initial concentration 100 mg/L, adsorbent dose 0.2 g, pH 9, temperature 30 °C, volume of solution 200 mL).

Figure 7 illustrates how the duration of contact time affected the elimination of the CV dye by the cross-linked starch. The highest elimination of the CV dye occurred within the initial 15 min, which is likely because of the availability of sorption sites at the start of the adsorption process. The removal efficiency peaked at 90% after 15 min, and this level was sustained with longer contact times. The obtained equilibrium time for CV adsorption in this study was lower than the equilibrium time of 60 min observed for a magnetically modified starch-based adsorbent reported in the literature [4]. A shorter equilibrium time is generally desirable in adsorption processes as it indicates faster adsorption kinetics and a more efficient utilization of the adsorbent material. Achieving equilibrium in a shorter duration can potentially translate into reduced processing times, improved throughput, and higher overall efficiency in practical applications such as wastewater treatment. The faster equilibrium time observed for the STMP cross-linked potato starch adsorbent can be attributed to several factors including the structural modifications induced by the cross-linking process, the presence of specific functional groups, and the potential for favorable interactions between the adsorbent and the CV dye molecules.

#### 2.2.3. Impact of Dye Initial Concentration

**Figure 8:**
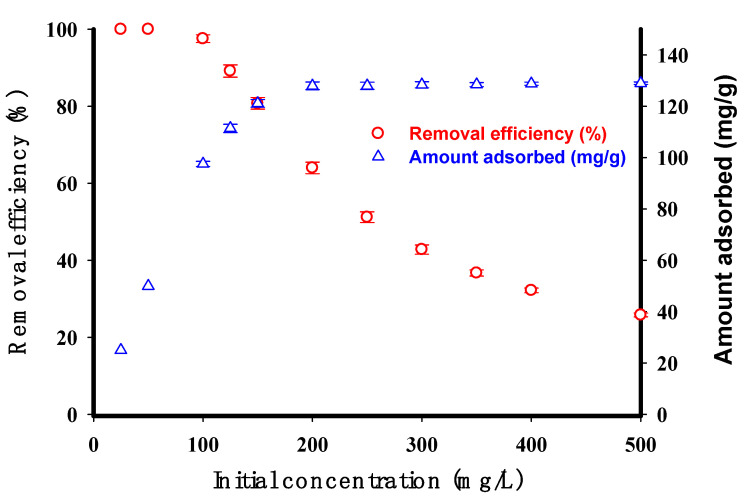
The initial concentration effect on the removal efficiency (%) as well as the adsorbed amount (mg/g) of CV by the cross-linked potato starch (initial adsorbent dose 0.2 g, time 90 min, pH 9, and temperature 30 °C, volume of solution 200 mL).

Figure 8 illustrates that an increase in the initial concentration of CV from 25 to 500 mg/L decreased its removal efficiency. Specifically, the removal efficiency remained at 100% until the initial concentration reached 50 mg/L, after which it gradually decreased as the concentration increased further. This can be explained by the fact that at lower dye concentrations, there are more available adsorption sites (not occupied) on the adsorbent surface. However, as more contaminants adsorb onto the surface, the available sites become saturated more quickly, leading to a decrease in the removal efficiency for CV pollutants. These results are in agreement with [38,39]. As depicted in Figure 8, a clear trend was observed wherein the adsorbed amount of CV increased with an increase in the initial CV concentration. Specifically, when the initial CV concentration was increased from 25 to 500 mg/L, a corresponding increase in the adsorption capacity was observed, ranging from 25 mg/g to 129 mg/g, respectively. This trend can be attributed to the higher driving force for mass transfer and the availability of more CV dye molecules in the solution at higher initial concentrations, facilitating enhanced adsorption onto the available active sites on the adsorbent surface [2,4,33]. However, the data also revealed that the adsorption capacity of the STMP cross-linked potato starch did not exhibit a dramatic change beyond an initial CV concentration of 200 mg/L. This observation suggests that the adsorbent approached its equilibrium adsorption capacity at higher initial CV concentrations, potentially due to the saturation of available adsorption sites on the adsorbent surface [2,4,33]. 

#### 2.2.4. Impact of Adsorbent Dose

**Figure 9:**
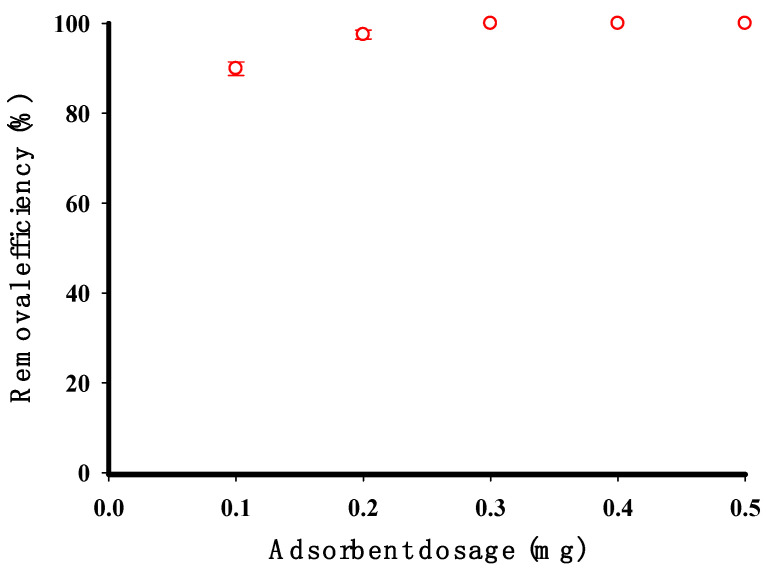
The adsorbent dosage impact on the removal efficiency of CV by the cross-linked potato starch (initial concentration 100 mg/L, contact time 90 min, pH 9, temperature 30 °C, volume of solution 200 mL).

Figure 9 shows how the amount of adsorbent used affects the removal of CV. Increasing the sorbent dosage from 0.1 to 0.5 g improved the elimination efficiency of the CV dye from 89.92% to 100%. Notably, 100% efficiency was achieved with a 0.2 g adsorbent dose. This improvement was due to the increased surface area and available adsorption sites [40]. Higher dosages of cross-linked starch also created a more significant concentration gradient between the CV solution and the adsorbent surface. This gradient promoted the movement of CV molecules from the bulk solution to the starch surface, increasing the chances of adsorption [41].

#### 2.2.5. Impact of Temperature

**Figure 10:**
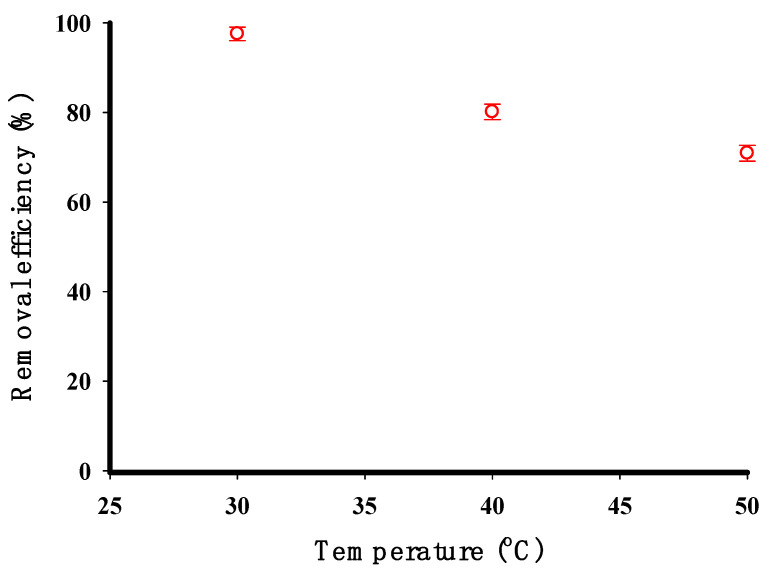
The impact of temperature on the elimination efficiency of CV by the cross-linked potato starch (initial concentration 100 mg/L, adsorbent dose 0.2 g, time 90 min, and pH 9).

Based on Figure 10, the highest removal efficiency of CV occurred at 30 °C, reaching 89.36%. As the temperature of the CV solution rose from 30 to 50 °C, the adsorption decreased, indicating a weak interaction of CV with the adsorbent surface [42]. The decrease in CV adsorption between 30 and 50 °C may be due to several factors such as the thermodynamic effects, solubility, diffusion, and alterations in the cross-linked starch structure [42]. These findings indicate that the adsorption of CV by the starch adsorbent is an exothermic process [4]. As the temperature rises, the boundary layer thickness decreases because CV is more likely to detach from the adsorbent surface and return to the solution, which results in reduced adsorption. Higher temperatures can also increase the solubility of CV, causing more dye molecules to stay in the liquid phase rather than attaching to starch surfaces. Additionally, higher temperatures can accelerate the movement of CV molecules through the solution, decreasing their interaction with the cross-linked starch surface and limiting adsorption. Furthermore, elevated temperatures can alter the properties and structure of starch. For more details, the enthalpy (ΔH°) and entropy (ΔS°) changes were calculated from the slope and intercept of the Van ‘t Hoff plot, respectively, as described in previous research [2,27,33]. The negative value of ΔH° (−112.8 kJ/mol) indicates that the overall adsorption process is exothermic in nature. This exothermic behavior suggests that the interactions between the CV molecules and the STMP cross-linked potato starch adsorbent as well as any structural or conformational changes in the adsorbent upon adsorption release energy to the surroundings. The exothermic nature of the adsorption process can be attributed to several factors such as the strong electrostatic attractions between the positively charged CV molecules and the negatively charged phosphate groups introduced by STMP crosslinking as well as hydrogen bonding interactions between the dye molecules and the functional groups on the adsorbent surface. On the other hand, the positive value of ΔS° (377.2 J/mol K) suggests that the adsorption process is accompanied by an increase in randomness or disorder. This increase in entropy can be attributed to the redistribution of CV molecules from the bulk solution onto the adsorbent surface, leading to a more disordered arrangement of the dye molecules on the adsorbent.

### 2.3. Possible Mechanism

The crosslinked starch network possesses various functional groups such as hydroxyl (–OH), ether (–O–), and phosphate (–PO_3_H–) groups. These functional groups can interact with the CV dye molecules through electrostatic interactions, hydrogen bonding, and other mechanisms. Notably, the presence of negatively charged phosphate groups introduced by STMP crosslinking facilitates the adsorption of positively charged CV molecules through strong electrostatic attractions. The formation of phosphate ester crosslinks through the SN_2_ (bimolecular nucleophilic substitution) reaction mechanism reinforces the starch network, enhancing its adsorption properties. In this reaction, the trimetaphosphate anion, (PO3)^3−^, acts as the nucleophile, and the hydroxyl groups (–OH) of the starch molecules act as the electrophilic centers or leaving groups. This mechanism reaction is favored under alkaline conditions.

### 2.4. Fuzzy Regression Results

**Figure 11:**
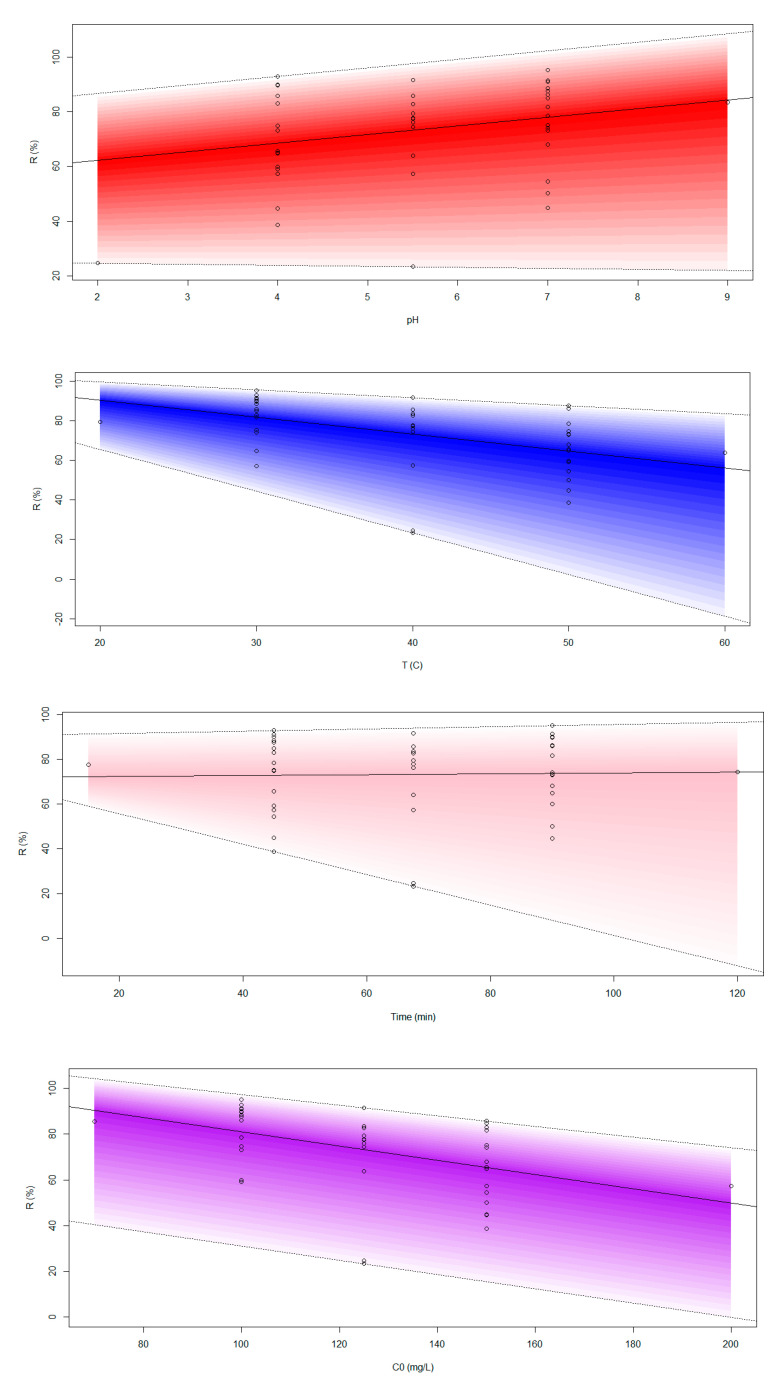

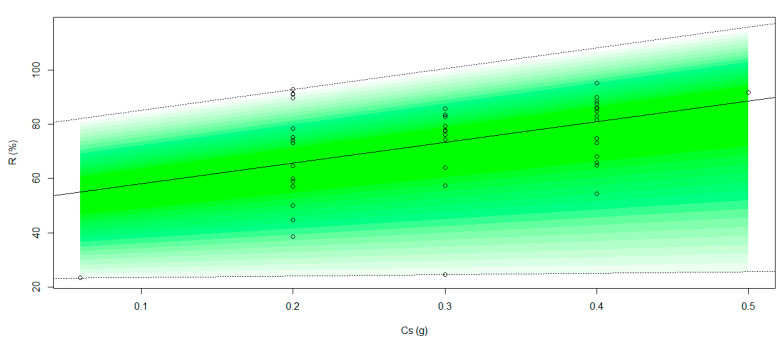
Changes in the effective parameters on removal efficiency by fuzzy regression.

Based on the fuzzy concept that describes changes within a range, the effect of changes in each of the variables is displayed on the pollutant removal percent (R) within a specific range. In Figure 11, the changes in the percentage of removal of crystal violet dye with respect to each parameter are depicted with three lines in the colored range. The central line represents simple regression, while the upper and lower lines represent the upper and lower bounds. The general equations of the three lines are listed below including the coefficients of all five independent variables.

The central tendency of the fuzzy regression model:

%R = 107.4098 + 4.095 × pH − 0.9228 × Temp − 0.0079 × time − 0.3356 × C_0_ + 76.1901 × C_s_

The lower boundary of the model support interval:

%R = 107.4047 + 4.095 × pH − 1.125 × Temp − 0.3492 × time − 0.3356 × C_0_ + 65.1358 ×C_s_

The upper boundary of the model support interval:

%R = 122.7789 + 4.095 × pH − 0.9228 × Temp − 0.0079 × time − 0.3356 × C_0_ + 76.1901 × C_s_

Based on the parameter coefficients in all three equations, positive coefficients were found for pH and cross-linked starch adsorbent dose, whereas negative effects were found for time, temperature, and initial concentration of CV pollutants in all three equations, which confirms the laboratory findings, aligning with previous research [23]. These values imply that the amount of CV dye pollutant removal from an aqueous solution is mainly influenced by the dosage of starch adsorbent. Following this, the influencing factors include pH, temperature, initial concentration of dye, and contact time. 

As seen in Figure 11, increasing the pH of the solution enhanced the percentage of dye elimination. In all three equations, the pH coefficient was constant, that is, increasing the pH by one unit increased the CV removal efficiency by an average of 4.095 units. As the temperature increased, the dye removal percentage decreased. For every one unit increase in temperature, on average, the removal percentage decreased between −0.9228 and −1.125. Additionally, for each unit of time increase, the minimum and maximum declines in the removal percentage were −0.0079 and −0.3492, respectively. An increase of one unit in the initial concentration of the pollutant in the solution led to a corresponding decrease in the removal percentage by 0.2 units. The range of change in pollutant removal percentage was wider with high temperatures and times than with the initial concentrations of higher pollutants. An increase in adsorbent dose led to a proportional increase in the pollutant removal percentage. The minimum and maximum removal values were 65.1358% and 76.1901%, respectively.

**Figure 12:**
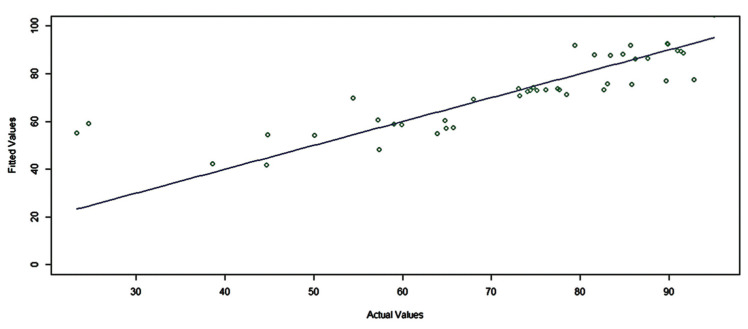
The 1:1 line plot illustrating the relationship between the experimental data and the predicted outcome of the fuzzy regression model.

The effectiveness of fuzzy regression in modeling the elimination of CV dye from an aqueous medium using starch is demonstrated by the plot of predicted values against the measured data in the lab (Figure 12). The high accuracy of fuzzy regression in this process is illustrated by the 1:1 line that fits between the predicted values and the measured data as well as the high value of the b coefficient (0.998) and the high R-squared value of 0.985 in Table 1. This alignment suggests that the fuzzy regression model effectively captures the behavior of the system. The high coefficient of b indicates a strong linear relationship between the predicted and measured values. The value of R-squared signifies that approximately 98.5% of the variability in the response variable (CV dye removal) can be explained by the model. In summary, fuzzy regression proves to be a robust method for modeling CV dye removal using starch, providing accurate predictions and strong statistical performance.

## 3. Materials and Methods

### 3.1. Preparation of Adsorbent

Potato starch powder was obtained from Merck Chemical Co., Darmstadt, Germany. Starch was cross-linked using a method proposed elsewhere [29,30]. Briefly, 10 g of starch was added to 100 mL of water that included 1 g Na_2_CO_3_ (10 g/100 g dry starch) and 2.5 g NaCl (25 g/100 g dry starch). While stirring, a crosslinking agent (1 g of sodium trimetaphosphate, STMP) was dissolved in the starch-containing slurry. The mixture was magnetically stirred at 200 rpm for 2–3 h at 40–45 °C in a water bath. The pH of the slurry was adjusted to 6.5 by adding 1 M hydrochloric acid. After the solid portion precipitated, we discarded the supernatant and washed the solid portion with water. The mixture was centrifuged to separate the cross-linked starch from the liquid phase. Finally, the STMP cross-linked starch was dried at 50 °C in an oven. Multiple techniques were employed to examine the characteristics of the adsorbent. 

### 3.2. Analytical Techniques

The Barret–Joyner–Halenda (BJH) and Brunauer–Emmett–Teller (BET) procedures were utilized to ascertain the sorbent’s pore-size distribution and specific surface area, respectively. Before analysis, the adsorbent sample is typically degassed to remove any moisture, impurities, or adsorbed gases from the porous structure. After the pretreatment, the degassed sample is cooled to cryogenic temperatures, usually using liquid nitrogen, to facilitate the adsorption of nitrogen gas onto the sample’s surface. The sample is then exposed to varying pressures of nitrogen gas, and the amount of gas adsorbed onto the sample is measured at each pressure point. After reaching a certain maximum pressure, the process is reversed, and the pressure is gradually reduced. The analysis of the sample with scanning electron microscopy (SEM) and energy-dispersive X-ray spectroscopy (EDX) was conducted using a Tescan microscope, model Vega3. The adsorbent sample was carefully mounted on a specialized holder, commonly known as a stub, and subsequently coated with a metal such as gold or platinum. The mounted and coated sample was then introduced into the SEM chamber, which was maintained under high vacuum conditions. The sorbent’s infrared absorption was acquired on KBr pellets using a Perkin Elmer FTIR Model: Spectrum RXI. The adsorbent sample was mixed with potassium bromide (KBr), which is an infrared-transparent salt. The homogeneous powder mixture was then subjected to high pressure, typically using a hydraulic or mechanical press, to form a transparent pellet. The prepared pellet was then carefully loaded into the sample holder or compartment of the FTIR spectrometer. The XRD study of the adsorbent was performed with a Bruker D8 Advance with Cu-Ka over the 2θ range of 5–85°. The powdered adsorbent sample was carefully loaded into a sample holder within the XRD instrument. As the X-rays interact with the crystalline structure of the adsorbent material, they were diffracted at specific angles in a range of 2θ between 5° and 85°. The initial solution pH was adjusted using 0.1 M HCl/NaOH.

### 3.3. Pollutant

**Figure 13:**
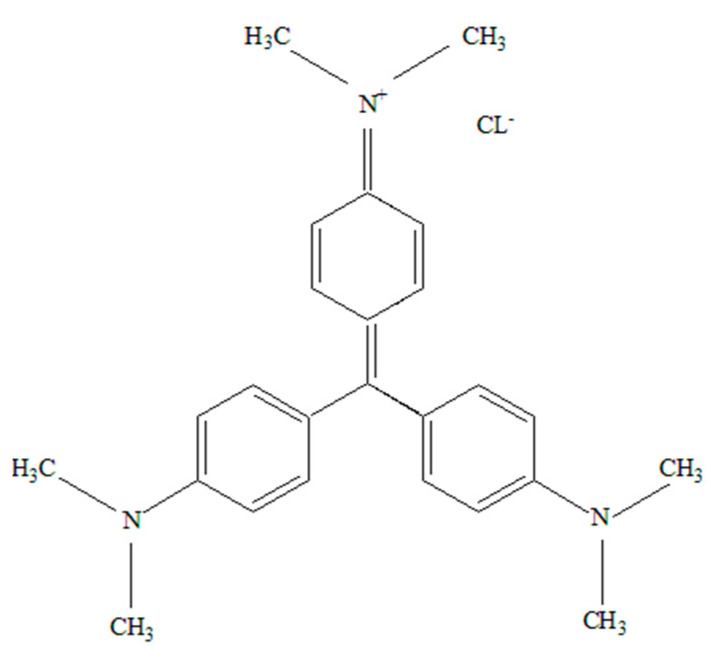
Structure of crystal violet.

The CV in this study was provided by Merck Chemical Co., Darmstadt, Germany. The molecular formula of CV is C_25_H_30_ClN_3_, and its molecular weight is 407.99 g/mol. It has a λ_max_ of 590 nm [4] and was used without requiring any further purification. The chemical structure of crystal violet is shown in Figure 13. The dye was dissolved in 1000 mL of distilled water to create a stock solution, which was then diluted to acquire working solutions ranging from 25 mg/L to 500 mg/L.

### 3.4. Adsorption Test

Batch sorption experiments were conducted to investigate the influence of the miscellaneous variables on the efficiency of CV removal. The studied parameters included pH values ranging from 2 to 9, temperature between 20 and 60 °C, contact time ranging from 15 to 120 min, initial CV dye concentration between 70 and 200 mg/L, and a cross-linked starch adsorbent dosage ranging from 0.06 to 0.5 g. Throughout the experiments, a consistent ratio of 0.2 g of modified starch was added to 200 mL of dye solution, resulting in an adsorbent dosage to solution ratio of 1 g/L. This ratio was maintained consistently across the experiments, except when the study explicitly aimed to evaluate the impact of varying adsorbent dosages on the adsorption process.

The experiments were carried out in 200-mL Erlenmeyer flasks containing dissolved dye and the adsorbent. The flasks were shaken with an orbital shaker operating at 120 rpm. After allowing enough time for the adsorption process, the adsorbent was separated from the solution using filtration. The residual dye concentration in the supernatant was specified using a UNICO-2100 UV–Vis spectrophotometer (Perkin-Elmer 3030 instrument) at the maximum CV absorption wavelength of 590 nm. The following formula computed the percentage of dye removal (*R*):

(1) $$R=\frac{C_{0}-C_{e}}{C_{0}}\times 100$$

where *C*_0_ and *C_e_*, are the initial and equilibrium CV concentrations (mg/L), respectively. 

### 3.5. Fuzzy Regression Modeling

In this study, a fuzzy regression technique was employed to model the adsorption of CV dye by cross-linked starch adsorbents. Fuzzy regression is a type of regression analysis that incorporates fuzzy concepts to handle uncertainties and imprecisions in the data [43]. The model was developed using the fuzzy least-squares method proposed by [44] to provide a range-based representation of the system’s behavior. The model’s output includes upper and lower bounds for the percentage of dye removal (*R*) based on the independent variables (pH, temperature, contact time, initial dye concentration, and adsorbent dosage).

The fuzzy regression model’s general form is given by the equation:

(2) $$R=a_{0}+a_{1}pH+a_{2}Temp+a_{3}time+a_{4}C_{0}+a_{5}C_{s}$$

where *a*_0_, *a*_1_, *a*_2_, *a*_3_, *a*_4_, and *a*_5_ are the coefficients needed to be determined. These coefficients were estimated using the fuzzy least-squares technique, which minimizes the fuzzified sum of squared residuals. The model provides three equations: one for the central tendency, and two for the upper and lower bounds of the model support interval.

## 4. Conclusions

The fuzzy regression modeling approach employed in this study offers a novel and more precise method for understanding and predicting the adsorption of CV from aqueous solutions. This study utilized cross-linked potato starch as the adsorbent, and this approach was determined to be more effective compared to traditional linear models that have been applied in previous similar investigations. The adsorption study accounted for various parameters such as pH levels ranging from 2 to 9, initial CV concentrations between 25 and 500 mg/L, contact time from 0 to 90 min, temperature between 30 and 50 °C, and adsorbent dose ranging from 0.1 to 0.5 g. Batch adsorption experiments demonstrated that the maximum elimination of CV was achieved at a pH of 9. The adsorbent reached equilibrium with the CV dye after 15 min of contact time, and the maximum removal of CV was observed at 30 °C. The SEM characterization analysis revealed a dispersed texture with a combination of spherical and polyhedron shapes in the cross-linked starch structure. Furthermore, FTIR analysis of the molecular structure before and after the adsorption experiments confirmed that the cross-linked starch adsorbent effectively adsorbed the CV dye. The batch experiment analysis demonstrated that increasing the pH from 2 to 9 as well as increasing the adsorbent dosage from 0.1 to 0.3 both improved the removal of the dye. Specifically, the dye removal rates increased from 68.88% to 97.55% with the pH change, and from 89.92% to 100% with the adsorbent dosage increase. The fuzzy regression model was able to satisfactorily describe the adsorption process. Furthermore, sensitivity analysis revealed that the adsorption of CV onto the cross-linked starch adsorbent was more sensitive to changes in the amount of adsorbent used. This modeling approach has practical applications as it provides valuable insights into the variables that influence the adsorption process, thereby facilitating optimization efforts. The importance of both the experimental and modeling techniques enhances our understanding of starch’s potential in water treatment, and lays the groundwork for further investigations in this field. Investigating the influence of diverse contaminants on the performance of the cross-linked starch adsorbent could provide valuable insights into its versatility and suitability for water treatment applications. Additionally, it is recommended that column studies are conducted to assess the performance of the cross-linked starch in continuous flow systems and to evaluate its potential for real-world applications. Furthermore, it is important to investigate the regeneration and reuse potential of the cross-linked starch adsorbent in order to improve its sustainability and cost-effectiveness.

## Figures and Tables

**Table 1:** Results of the fuzzy regression model.

b	Std	t	*p*. Value	R^2^
0.9984	0.01672	0.09885	0.921	0.985

## Data Availability

Data will be made available on request.
